# Relationships between nine neuropsychiatric disorders and cervical cancer: insights from genetics, causality and shared gene expression patterns

**DOI:** 10.1186/s12905-024-03234-5

**Published:** 2024-07-08

**Authors:** Jie Li, Jie Qi, Junqin Zhang, Yuan Zhang, Xianghua Huang

**Affiliations:** 1 Department of Gynecology, Hebei General Hospital, Shijiazhuang, Hebei 050051 China; 2 Department of Obstetrics and Gynecology, Second Hospital of Hebei Medical University, No. 215, HePing West Road, Shijiazhuang, Hebei 050000 China

**Keywords:** Cervical cancer, Depression, Genetic correlation, Causal association, Gene expression patterns

## Abstract

**Background:**

Neuropsychiatric disorders and cervical cancer exert substantial influences on women’s health. Furthermore, neuropsychiatric disorders frequently manifest as common symptoms in cancer patients, potentially increasing the risk of malignant neoplasms. This study aimed to identify neuropsychiatric disorders that are genetically and causally related to cervical cancer and to investigate the molecular mechanisms underlying these associations.

**Methods:**

GWAS data related to nine neuropsychiatric disorders, namely, schizophrenia, bipolar disorder, autism spectrum disorder, Parkinson’s disease, anxiety, Alzheimer’s disease, mood disorders, depression, and alcohol dependence, were obtained to calculate heritability (*h*^2^) and genetic correlation (r_g_) with cervical cancer using linkage disequilibrium score regression (LDSC). Mendelian randomization (MR) analysis of the two cohorts was employed to assess the causal effects. Shared gene expression pattern analysis was subsequently conducted to investigate the molecular mechanism underlying these significant associations.

**Results:**

Anxiety, mood disorders, depression, and alcohol dependence were genetically correlated with cervical cancer (all adjusted *P* < 0.05). Only depression was causally related to cervical cancer in both the discovery (OR_IVW_: 1.41, *P*_IVW_ = 0.02) and replication cohorts (OR_IVW_: 1.80, *P*_IVW_ = 0.03) in the MR analysis. Gene expression pattern analysis revealed that 270 genes related to depression and cervical cancer, including tumour necrosis factor (TNF), were significantly upregulated in cervical cancer patients, while vascular endothelial growth factor A (VEGFA), transcription factor AP-1 (JUN), and insulin-like growth factor I (IGF-I) were associated with prognosis in cervical cancer patients (all *P* < 0.05). These overlapping genes implicated the involvement of multiple biological mechanisms, such as neuron death, the PI3K-Akt signalling pathway, and human papillomavirus infection.

**Conclusions:**

Genetic, causal and molecular evidence indicates that depression increases the risk of cervical cancer. The TNF, VEGFA, JUN, and IGF-1 genes and the neuron death, PI3K-Akt, and human papillomavirus infection signalling pathways may possibly explain this association.

**Supplementary Information:**

The online version contains supplementary material available at 10.1186/s12905-024-03234-5.

## Background

Cervical cancer is the fourth most prevalent malignancy affecting women worldwide, with nearly 604,000 new cases and an estimated 342,000 deaths documented in 2020, according to Global Cancer Statistics (GLOBOCAN) [1, 2]. Historically, the primary focus in understanding the pathogenesis and progression of cervical cancer has centred on persistent high-risk human papillomavirus (HPV) infection [3]. The widespread deployment of HPV vaccines among females has indeed marked a significant milestone in the reduction of HPV infections and advanced cervical cancer cases [4, 5]. However, it has become increasingly evident that the incidence and progression of cervical cancer are influenced by a spectrum of additional factors, including the use of oral contraception, smoking, multiple sexual partners, high parity, immune deficiencies, and early introduction to sexual intercourse [6–10]. Intriguingly, neuropsychiatric disorders, recognized as pivotal influencers of cancer incidence, recurrence, and mortality, have also emerged as being epidemiologically associated with cervical cancer. Among these, depression and anxiety, which are prevalent mental health conditions among women of childbearing age [11–13], have been more frequently identified in cervical cancer patients [14–16]. In addition, other psychiatric traits, such as alcohol dependence [17, 18], bipolar disorder and mood disorders [19], have been proposed as potential factors influencing the risk of cervical cancer.

Despite these intriguing observations, the existing genetic evidence and molecular mechanisms supporting the roles of these neuropsychiatric disorders in cervical cancer pathogenesis remain unknown. Genome-wide association studies (GWASs) have revealed a broad spectrum of genetic variants across the entire genome that are associated with complex diseases [20, 21]. Association analysis based on GWAS data can take advantage of the stable genetic structure established within large population cohorts to enhance the statistical power to detect genetic correlations between traits. GWAS data can also serve as a valuable resource for conducting Mendelian randomization analyses, a method that can discern the direction of causal effects [22].

**Fig. 1:**
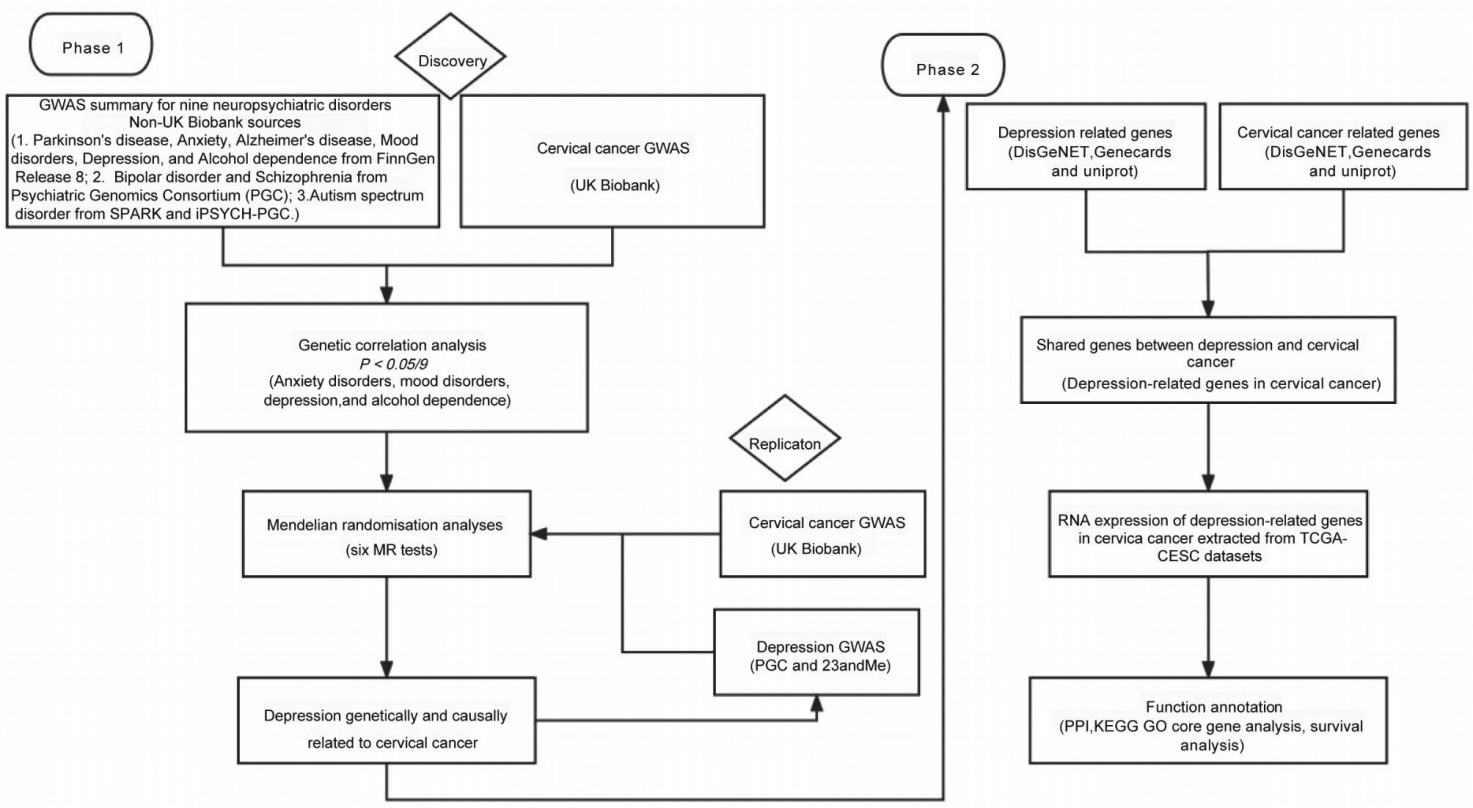
Flow chart of this study

As summarized in Fig. 1, our study was designed to provide a comprehensive understanding of how neuropsychiatric disorders influence cervical cancer susceptibility and progression, elucidating their relationships from genetics, causality and molecular perspectives.

## Methods

### Study design

The overall study design is illustrated in Fig. 1. In Phase 1, we gathered GWAS summary statistics for nine neuropsychiatric disorders from three non-UK Biobank sources (as detailed in Additional File 1 and the methods below) and for cervical cancer from the UK Biobank (6,563 cases and 410,350 controls). During the discovery phase, genetic correlations between these neuropsychiatric disorders and cervical cancer were assessed using linkage disequilibrium score regression (LDSC). Four neuropsychiatric disorders—anxiety disorders, mood disorders, depression, and alcohol dependence—were found to have significant genetic correlations with cervical cancer (adjusted P value < 0.05). We then performed two-sample Mendelian randomization (MR) analyses to explore the causal relationships between these four neuropsychiatric disorders and cervical cancer, identifying depression as the primary causal factor. To validate this finding, we utilized a large-scale GWAS meta-analysis of data from 23andMe and PGC for depression, along with cervical cancer GWAS data from the UK Biobank (1,889 European cases and 461,044 European controls), as a replication cohort. The replication phase confirmed the causal relationship between depression and cervical cancer through two-sample MR analysis.

In Phase 2, to further investigate the molecular mechanisms underlying this causality, we collected genes associated with depression and cervical cancer from the DisGeNET, GeneCards, and UniProt databases. We conducted shared expression pattern analysis, RNA differential expression analysis, functional annotation (including GO, KEGG enrichment, and PPI analyses), and survival analysis to elucidate the role of depression-related genes in cervical cancer.

### Data sources and preprocessing

The GWAS summary statistics datasets utilized in this study were obtained from five distinct sources (comprehensively detailed in Additional File 1). In the discovery stage, the cervical cancer GWAS included 6,563 cases and 410,350 controls [23] and was sourced from the UK Biobank (UKB), a well-established population-based study encompassing 500,000 individuals recruited from across the United Kingdom [24]. We also accessed GWASs for six neuropsychiatric disorders, Parkinson’s disease, anxiety, Alzheimer’s disease, mood disorders, depression, and alcohol dependence, from FinnGen Release 8 (release date: December 2022, accessed at https://r8.finngen.fi/) [25]. The FinnGen consortium is an important academic medicine project that combines clinical information with genomic data from 500,000 Finnish participants. Furthermore, GWASs for bipolar disorder (BD) and schizophrenia (SCZ), encompassing 53,555 patients (20,129 BD patients and 33,426 SCZ patients) and 54,065 controls, were obtained from the Psychiatric Genomics Consortium (PGC) [26]. The autism spectrum disorder (ASD) GWAS data originated from a meta-analysis involving the SPARK European population and iPSYCH-PGC, including 22,916 cases and 32,504 controls [27]. In the replication stage, summary-level GWAS statistics specific to cervical cancer involving 1,889 European cases and 461,044 European ancestry controls were downloaded from OpenGWAS in MRbase [28]. Considering that our replication stage primarily focused on MR analysis, which requires only the significant SNPs associated with exposure, we specifically extracted the significant SNPs associated with depression directly from the tables and supplementary files of a large-scale GWAS meta-analysis sourced from 23andMe and PGC [29]. We utilized the LiftOver tool to convert GWAS summary data (mainly FinnGen-derived GWAS data) from the reference genome GRCh38/hg38 to the GRCh37/hg19 format [30].

### Genetic correlation analysis using linkage disequilibrium score regression (LDSC)

In our research, we utilized the software LDSC v1.0.1 (accessed at https://github.com/bulik/ldsc) to calculate the observed-scale single-nucleotide polymorphisms (SNPs) heritability for each trait and to assess the global genetic correlations (r_g_) between the nine neuropsychiatric disorders in the discovery cohort and cervical cancer [31]. Before conducting the analysis, we excluded SNPs located within the major histocompatibility complex (MHC) region, a minor allele frequency (MAF) of less than 1%, and an imputation quality score of less than 90% using the munge_sumstats command to ensure the reliability of the results. Following these rigorous data processing steps, high-quality GWAS summary statistics were merged with a HapMap3 reference panel to reduce statistical noise and ensure robust genetic correlation analysis. The commands ldsc_h2 and ldsc_h2 were used to compute heritability and genetic correlations, respectively.

### MR analysis

Eligible instrument variables (IVs) for MR analysis were selected from the GWAS summary statistics according to the following criteria. First, using a *P* value threshold of 5 × 10^− 8^, we extracted the SNPs correlated with neuropsychiatric disorders at the genome-wide significance level. Subsequently, the clumping procedure was performed by PLINK (version: 1.9) [32] to remove SNPs in linkage disequilibrium (LD) using the European panel of the 1000 Genomes Project, with parameters including an LD threshold of r^2^ < 0.1 and a clumping window size of 500 kb. Finally, we excluded palindromic SNPs and SNPs missing from the cervical cancer GWASs through harmonization. After these quality control steps, the remaining SNPs were regarded as eligible IVs. Next, MR‒Egger regression analysis was used to eliminate the confounding effect of some IVs possessing horizontal pleiotropy [33]. Moreover, Cochran’s Q statistic was calculated to assess the heterogeneity among SNPs [34]. For the sensitivity analysis, a leave-one-out test was employed to exclude the bias of causal inference from a single SNP. Additionally, we calculated the F statistics for each SNP to rule out weak IV bias (F > 10 indicating no weak IV bias). Two-sample Mendelian randomization (TSMR) analysis was performed in R 4.2.2 software with the R package “TwoSampleMR” (version 0.5.6). In total, we utilized six MR models to estimate the causal effect of psychiatric traits on cervical cancer, including MR‒Egger, inverse-variance weighted (IVW), mixed random effects IVW (IVW-mre), weighted median, weighted mode and simple mode. IVW can delineate a weighted regression of the SNP-outcome effect on SNP-exposure with an intercept at zero [35]. The IVW-mre model should be the ideal method for causal inferences if apparent heterogeneity is present [36]. The MR‒Egger method can provide a more conservative causal inference away from the confounding effect of pleiotropy [37]. The weighted median method will generate a robust causal effect judgement even in the presence of more than 50% invalid IVs [36]. Moreover, when the majority of IVs are invalid, we can conduct a weighted mode test to make a reliable causal inference [38]. The significance thresholds of all the above tests were set to 0.05. In most cases, IVW was the primary choice to assess the causal effect of neuropsychiatric disorders on cervical cancer.

### Identification of depression-related genes in cervical cancer

To identify genes that may play a role in both depression and cervical cancer, we conducted an extensive analysis using several reputable databases, including the DisGeNET (https://www.disgenet.org/; accessed on 25 June 2023) [39], GeneCards (https://genecards.weizmann.ac.il/v3/; accessed on 25 June 2023) [40], and UniProt (https://www.uniprot.org/; accessed on 25 June 2023) [41]. These databases provided valuable gene data relevant to our investigation. For depression-related genes, we conducted searches in DisGeNET using the term “Mental Depression, C0011570” and filtered results with a GDA score > 0.1. We also retrieved data from GeneCards using the term “Depression” and filtered the data by a relevance score > 5. Additionally, we retrieved data from UniProt using the term “Depression” and retained genes specific to humans. For cervical cancer-related genes, our search strategy involved using DisGeNET with the term “Cervical cancer, C4048328” and filtering results with a GDA score > 0.1. We also retrieved data from GeneCards using the term “Cervical cancer” and filtered the data by a relevance score > 5. Furthermore, we retrieved data from UniProt using the term “Cervical cancer” and retained genes specific to humans. This search strategy ensures consistency in the gene sets obtained by researchers. Eventually, we identified the genes that were common to both depression and cervical cancer, forming a gene set specific to the intersection of these two conditions. These genes were categorized as depression-related genes in cervical cancer.

### Functional enrichment analysis

Subsequently, we utilized the GWAS catalogue (https://www.ebi.ac.uk/gwas/) and the dbSNP database (https://www.ncbi.nlm.nih.gov/snp/) to map the eligible IVs identified in both the discovery and replication cohorts to genes. The RNA expression matrix and clinical information of The Cancer Genome Atlas-Cervical squamous cell carcinoma and endocervical adenocarcinoma (TCGA-CESC) dataset, including 304 cervical cancer samples and 3 paired normal tissues, were retrieved and qualified using the GDCRNATools R package [42]. DESeq2 was used to identify differentially expressed genes (DEGs), utilizing an adjusted *P* value threshold of < 0.05 and a log fold change (logFC) cut-off of > 1. To further annotate the mapped genes and depression-related genes in cervical cancer, we performed Gene Ontology (GO) and Kyoto Encyclopedia of Genes and Genomes (KEGG) pathway analyses using the R packages “clusterProfiler” and “org.Hs.eg.db”. This analysis provided insights into the functional characteristics and biological pathways associated with the identified genes. Furthermore, we constructed protein‒protein interaction (PPI) networks based on the mapped genes and depression-related genes in cervical cancer. To identify the most influential genes within the PPI networks, we applied cytoHubba’s maximal clique centrality (MCC) method in Cytoscape 3.9.1 to determine the top 10 hub genes. These hub genes represented highly interconnected and functionally significant nodes within the network. We proceeded to overlap the identified DEGs with these hub genes. Additionally, we conducted survival analysis on these hub genes using the TCGA-CESC datasets with the survival and survminer R packages.

## Results

### Genetic correlation between nine neuropsychiatric disorders and cervical cancer

**Fig. 2:**
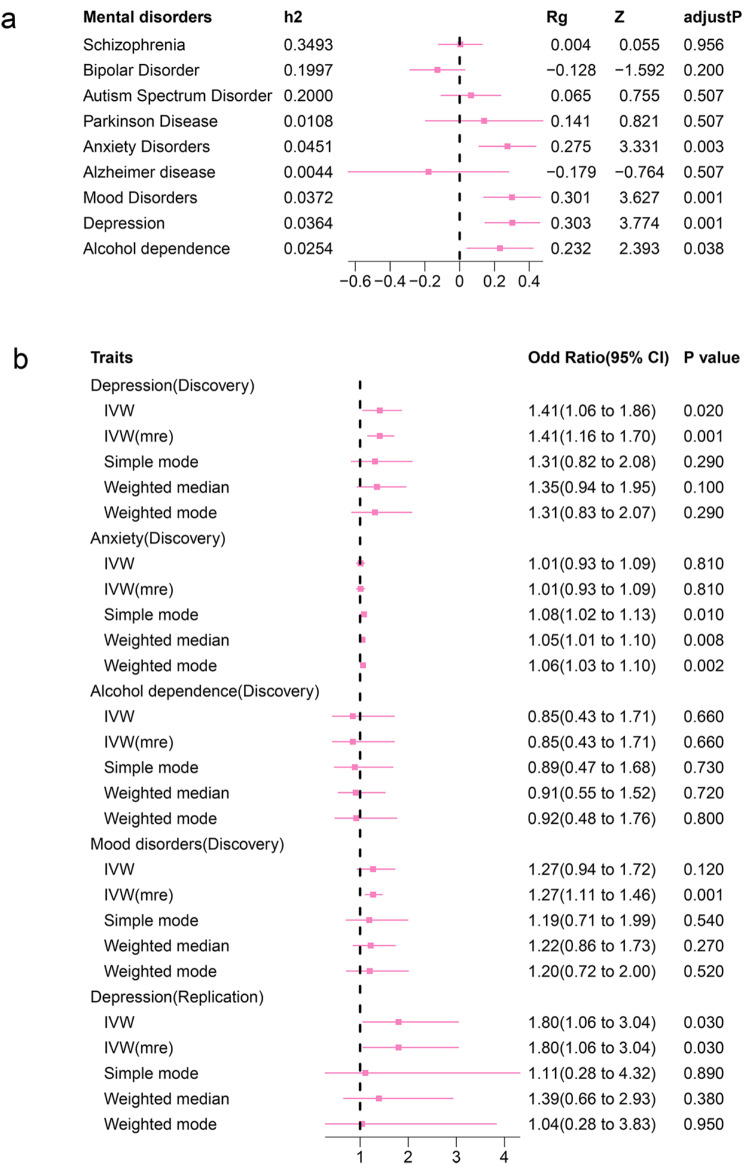
Summary of genetic correlation and Mendelian randomization results

We compiled publicly available GWAS summary statistics related to nine neuropsychiatric disorders, namely, schizophrenia, bipolar disorder, autism spectrum disorder, Parkinson’s disease, anxiety, Alzheimer’s disease, mood disorders, depression, and alcohol dependence. After performing heritability (*h*^2^) and genetic correlation (r_g_) calculations using these summary statistics, notable patterns emerged. Specifically, we observed that anxiety (*h*^2^ = 0.004, r_g_ = 0.275, adjusted *P* = 0.03), mood disorders (*h*^2^ = 0.037, r_g_ = 0.301, adjusted *P* = 0.001), depression (*h*^2^ = 0.036, r_g_ = 0.303, adjusted *P* = 0.001), and alcohol dependence (*h*^2^ = 0.025, r_g_ = 0.232, adjusted *P* = 0.038) exhibited genetic similarity with cervical cancer (as depicted in Fig. 2a). In contrast, SCZ, BP, ASD, Parkinson’s disease, and Alzheimer’s disease did not demonstrate statistically significant correlations with cervical cancer (adjusted *P* > 0.05).

- Forest plots summarized the genetic correlations between cervical and nine neuropsychiatric disorders, that indicated depression, anxiety, mood disorders and alcohol dependence were genetically correlated with cervical cancer with statistical significance.
- Mendelian randomization results by 5 MR tests (IVW, IVW-mre, Simple mode, Weighted median, and Weighted mode) in both discovery and replication cohort, and only depression was causally related to cervical cancer (*P* < 0.05).

### Genetic instrumental variables for MR analysis

Four neuropsychiatric disorders with evidence of a notable genetic correlation with cervical cancer were included for further MR analysis. After several screening processes, a total of 54 SNPs were obtained as eligible genetic IVs in the MR analysis, including 8, 19, 6, and 6 SNPs for depression, anxiety, alcohol dependence, and mood disorders, respectively, in the discovery cohort and 15 SNPs for depression in the replication cohort (Table 1). Furthermore, the F-statistic for each SNP was greater than ten, suggesting that our MR analysis was free from the influence of weak IV bias (Additional file 2). According to the Cochran’s Q test (IVW method), heterogeneity existed among the SNPs related to anxiety (Q-statistic = 129.53, *P* < 0.05) and alcohol dependence (Q-statistic = 21.30, *P* < 0.05) in the discovery cohort (Table 1); therefore, IVW-mre was the optimal method for explaining the causal associations between these two psychiatric traits and cervical cancer. Moreover, MR‒Egger analysis revealed no apparent horizontal pleiotropy for any of the traits (*P* > 0.05) (Table 1).

**Table 1:** Summary of eligible IVs selection, horizontal pleiotropy and heterogeneity test

Traits	Eligible IVs	Q-statistic (IVW)	Q-pval	Intercept (MR-Egger)	Pleiotropy_pval
Depression (Discovery)	8	3.30	0.86	0.06	0.39
Anxiety (Discovery)	19	129.53	6.53E-19	-0.1	0.09
Alcohol dependence (Discovery)	6	21.30	0.0007	0.08	0.59
Mood disorders (Discovery)	6	1.03	0.96	0.04	0.80
Depression (Replication)	15	14.33	0.43	-0.02	0.74

### Putative causality between depression and cervical cancer confirmed in discovery and replication cohorts

**Fig. 3:**
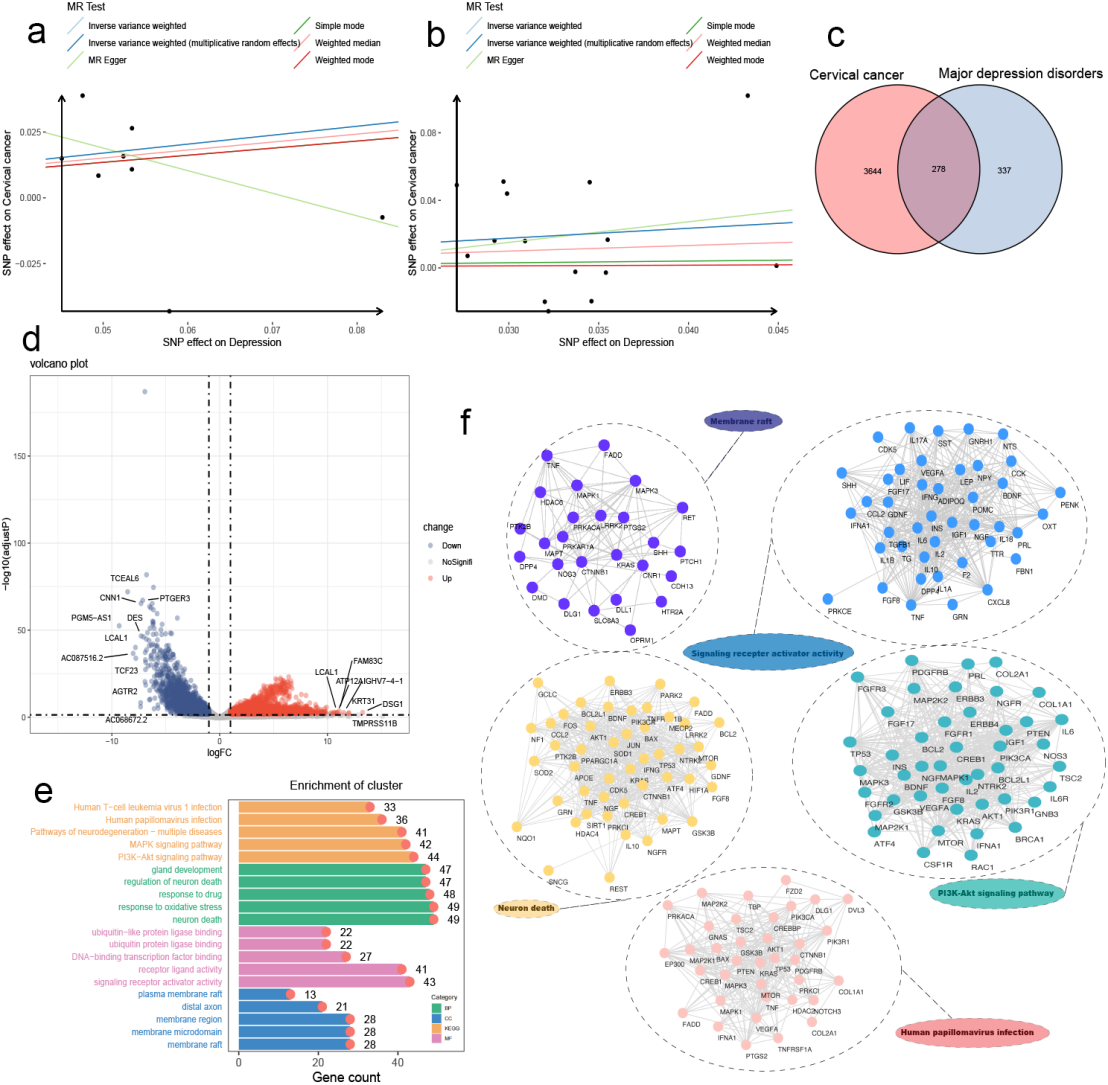
Function annotation based on genes associated with depression in cervical cancer. **a-b**. Scatter plots illustrated the effects of instrumental variables on cervical cancer and depression, which were demonstrated to be causally related in our study. **c**. Venn plot identified the genes related to major depression disorders in cervical cancer. **d**. Volcano plot visualized the gene expression changes between cervical cancer tissues and normal controls based on TCGA-CESC datasets, red dots represented upregulated genes and blue dots represented downregulated genes. **e**. Bar graph was generated to visualize the top 5 terms in each section, including biological process (BP) terms, cell component (CC) terms, molecular function (MF) terms, and KEGG signaling pathways. **f**. Protein-Protein Interaction networks of the critical terms with the highest enrichment scores or counts in each section were conducted to enhance our understanding of the molecular mechanisms and interactions underlying the observed enrichments

The causal effects of four neuropsychiatric disorders on cervical cancer were evaluated by six MR methods, including IVW, IVW-mre, MR‒Egger, simple, weighted median, and weighted mode. Figure 3a shows the MR results for 5 methods, excluding the MR‒Egger test, as some MR‒Egger test results deviated substantially from those of the other methods (Additional file 3). In the discovery cohort, depression was significantly causally associated with cervical cancer (OR_IVW_: 1.41, 95% CI: 1.06–1.86; *P*_IVW_ = 0.02). The other three traits, anxiety (OR_IVW−mre_: 1.01, 95% CI: 0.93–1.09; *P*_IVW−mre_ = 0.81), alcohol dependence (OR_IVW−mre_: 0.85, 95% CI: 0.43–1.71; *P*_IVW−mre_ = 0.66), and mood disorders (OR_IVW_: 1.27, 95% CI: 0.94–1.72; *P*_IVW_ = 0.12), were not significantly associated with cervical cancer (as summarized in Fig. 2b). In the replication cohort, the leave-one-out analysis suggested that rs11209948 can substantially modify the overall causal effect (OR_IVW_: 1.60, *P* = 0.08). After omitting rs11209948, depression was also demonstrated to be significantly associated with cervical cancer (OR_IVW_: 1.80, 95% CI: 1.06–3.04, *P* = 0.03). The scatter plots in Fig. 3a and b were constructed to illustrate the causal effect of the IVs for depression on cervical cancer in both cohorts. Scatter plots for the remaining three mental traits are provided in Supplementary Fig. 1 and available in Additional file 4. Additionally, the results of the leave-one-out analyses are represented using a forest plot in Supplementary Fig. 2, which can be found in Additional file 4.

### Functional annotation of genes associated with depression in cervical cancer

In previous analyses, we identified a total of 23 genetic instrumental variables to clarify the causal relationship between depression and cervical cancer (8 SNPs from the discovery cohort and 15 SNPs from the replication cohort). We mapped the 23 eligible IVs to 22 genes via the GWAS catalogue and dbSNP database (refer to Additional file 5).

To explore potential genes associated with cervical cancer onset and progression, we collected a total of 4,020 candidate genes from DisGeNET (38 genes with GDA score > 0.1), GeneCards (3,844 genes with relevance score > 5), and UniProt (138 reviewed genes). Similarly, for genes related to depression, we obtained a list of 780 candidate genes from DisGeNET (287 genes with GDA score > 0.1), GeneCards (415 genes with relevance score > 5), and UniProt (78 reviewed genes). The compiled list of genes related to depression and cervical cancer can be found in Additional file 6. By creating a Venn diagram (Fig. 3c), we identified 278 overlapping genes as depression-related genes in cervical cancer.

Furthermore, we downloaded TCGA-CESC datasets and performed differential expression gene analysis. By applying the criteria of an adjusted *P* value < 0.05 and logFC > 1, we identified 2,495 downregulated genes and 2,947 upregulated genes. Subsequently, we merged the 22 mapped genes and the 278 depression-related genes in cervical cancer with the TCGA-CESC RNA expression matrix (Fig. 3d), which resulted in an RNA expression matrix of 270 genes associated with depression in cervical cancer.

To gain further insight, we conducted GO and KEGG pathway enrichment analyses, which revealed 3,294 statistically significant biological process (BP) terms, 172 cell component (CC) terms, 225 molecular function (MF) terms, and 190 signalling pathways (*P* < 0.05). The top 5 terms in each category were visualized using a bar graph (Fig. 3e). Notably, the critical terms with the highest enrichment scores or counts in each section were neuron death (GO_BP_:0070997), membrane raft (GO_CC_:0045121), signalling receptor activator activity (GO_MF_:0030546), the PI3K-Akt signalling pathway (hsa04151), and human papillomavirus infection (hsa05165) (Fig. 3f). A comprehensive summary of all significant enrichment terms can be found in Additional file 7.

### Identification of hub genes and prognostic markers related to depression in cervical cancer

**Fig. 4:**
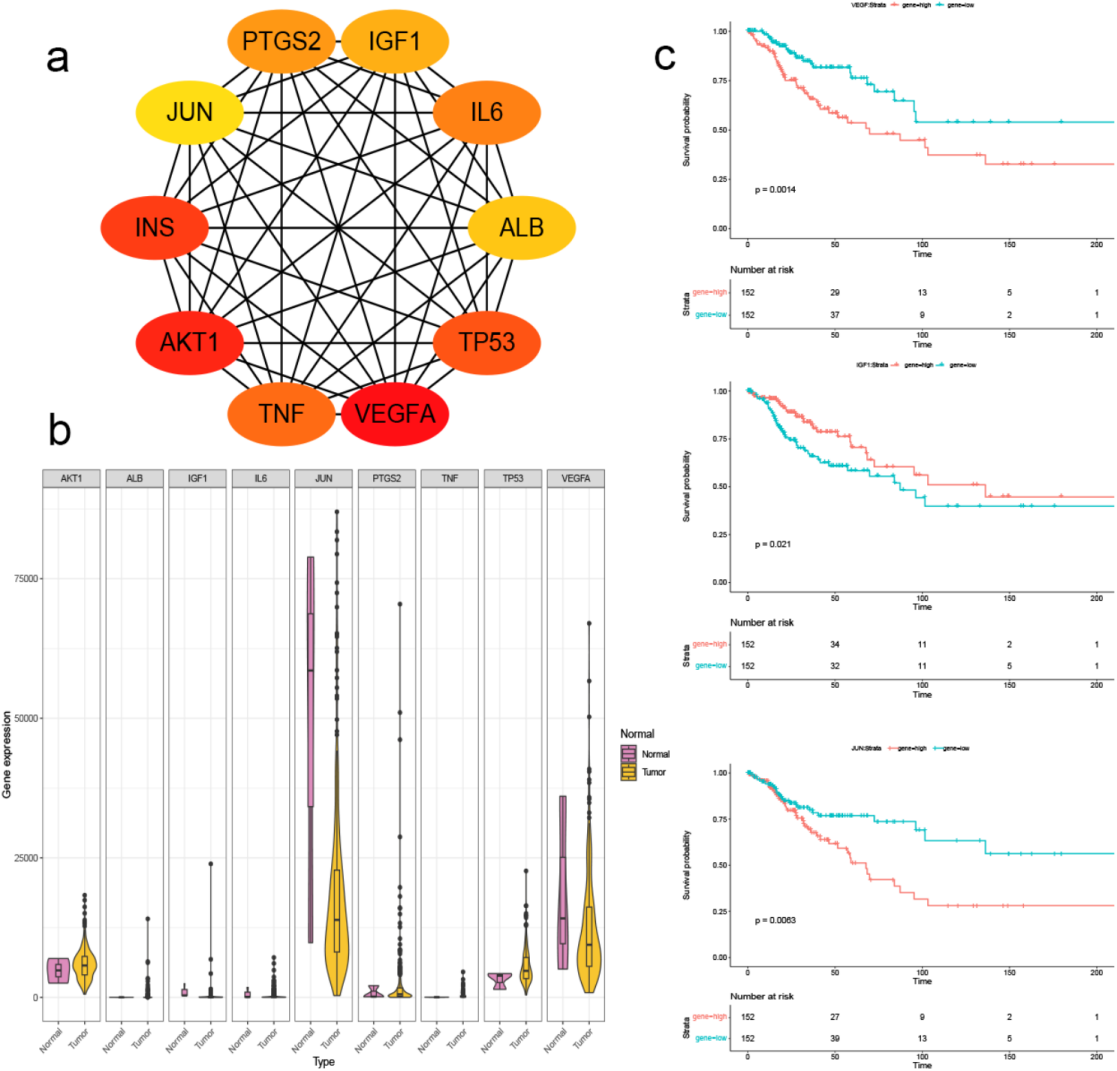
Identification of hub genes and prognostic markers related to depression in cervical cancer. **a**. Protein-Protein Interaction network provided a comprehensive view of the interactions and relationships among 10 hub genes. **b**. The violin plots were used to depict the expression profiles of the hub genes in the TCGA-CESC dataset. **c**. Survival curves revealed that decreased expression of VEGFA (*P* = 0.0014), JUN (*P* = 0.0063), and elevated expression of IGF1 (*P* = 0.021) were associated with improved prognosis

In parallel, a PPI network was constructed with 270 genes related to depression in cervical cancer patients. Using the MCC mode in Cytoscape 3.9.1, we successfully identified 10 hub genes within this network (Fig. 4a). To gain insights into the expression profiles of these hub genes in cervical cancer, we generated violin plots. Remarkably, our analysis revealed significant upregulation of the tumour necrosis factor (TNF) gene (*P* = 0.037) (Fig. 4b).

Furthermore, survival analysis was performed for these 10 hub genes. The results revealed that decreased expression of vascular endothelial growth factor A (VEGFA) (*P* = 0.0014) and transcription factor AP-1 (JUN) (*P* = 0.0063) and elevated expression of insulin-like growth factor I (IGF-I) (*P* = 0.021) were associated with improved prognosis in cervical cancer patients (Fig. 4c).

## Discussion

In our study, we identified four neuropsychiatric disorders (anxiety, mood disorders, depression, and alcohol dependence) that are genetically linked to cervical cancer. However, only depression consistently demonstrated a causal relationship with cervical cancer in both the discovery and replication cohorts. Ultimately, shared gene expression pattern analysis yielded molecular insights to elucidate the positive associations.

In our MR analysis, we took deliberate steps to minimize sample overlap between the exposure and outcome by selecting samples from separate cohorts. As a result, the heritability estimates reported in our study may appear lower than previously reported estimates for various neuropsychiatric disorders. However, despite these differences, we were able to validate the genetic association and establish causality between depression and cervical cancer. According to previous cohort studies, depression, one of most prevalent cancer-related symptoms, is frequently reported to be associated with cervical cancer, with mixed results. In one 19-year prospective cohort study, a negative correlation was discovered between cervical cancer and depression in 7860 women (HR 0.90, 95% CI 0.83, 0.98) [43]. In another Chinese prospective cohort, shorter overall survival was observed in cervical cancer patients with depression than in those without depression [44]. Similar to the findings of the second study, our results also supported that depression may be a risk factor for cervical cancer, with ORs of 1.41 in the discovery cohort and 1.80 in the replication cohort. Moreover, the results of several other studies indicated that anxiety and depression may be associated with advanced disease or decreased survival in cervical cancer patients [15, 44–47]. Overall, our findings add to the existing evidence and further support the potential link between depression and cervical cancer. The use of MR methods strengthens the causal interpretation of the observed associations, highlighting the importance of mental health factors in the context of cervical cancer.

Previous reports have highlighted the presence of genes shared between neuron death and colorectal cancer [48], suggesting a potential relationship between these processes. Our study, which identified neuron death as a significantly enriched term in the GO analysis, further supported the notion of a potential link between depression-related neurobiological processes and the development of cervical cancer. The PI3K-Akt signalling pathway, a well-known pathway involved in tumour cell growth, proliferation, apoptosis, metastasis, and survival, has been reported to play a crucial role in cervical cancer development [49]. Additionally, studies have indicated a potential link between depression and the dysregulation of this pathway [49]. Our findings provide further evidence supporting the role of the PI3K-Akt signalling pathway in the pathogenesis of both cervical cancer and depression. HPV infection has previously been hypothesized to be a potential mechanism through which depression influences cervical cancer risk [43]. The significant enrichment of “human papillomavirus infection” in our KEGG pathway analysis provides evidence for the potential association between HPV infection and depression in the development of cervical cancer.

The proinflammatory cytokine TNF plays a pivotal role in the immune response and inflammation and has been shown to be elevated in individuals with depression [50]. Elevated TNF expression may contribute to the interaction between cervical cancer and depression by promoting inflammation and disrupting cellular processes. Interestingly, VEGFA is also a risk factor for depression and can be utilized as a prognostic factor for the development of recurrent depression [51, 52]. Additionally, evidence has shown that moderate levels of IGF-1 are associated with a reduced risk of depression [53]. These findings highlight the potential interplay among these hub genes, cervical cancer prognosis, and the risk of depression, providing valuable insights for future research and therapeutic approaches.

Our study has several strengths that enhance the robustness of our findings. First, a large sample size from multiple datasets was utilized. Second, we employed rigorous methodology throughout our study, including LDSC, MR and shared gene pattern analysis. This approach ensures the reliability and reproducibility of our results, enhancing the validity of our conclusions. Furthermore, the integration of multiple data sources, such as GWAS data, RNA expression data, clinical information, and survival data, enables a holistic understanding of the molecular mechanisms underlying cervical cancer progression and prognosis. This multidimensional approach enriches our study and offers valuable insights into the complex nature of the disease.

Despite the significant contributions of our research, several limitations deserve attention. First, the disproportionate case‒control ratio observed in the cervical cancer GWAS, with the largest number of cases being less than 7,000, is constrained by the scarcity of relevant research. This limitation may affect the statistical power and generalizability of our findings, necessitating caution when interpreting the results. Second, while our study included comprehensive functional annotation, the lack of support from further experimental validation experiments may limit the reliability of the functional interpretations. Finally, our MR analysis did not account for sex disparities, which may impact the interpretation of causal relationships between exposures and outcomes.

## Conclusions

In conclusion, genetic, causal and molecular evidence indicates that depression increases the risk of cervical cancer. The TNF, VEGFA, JUN, and IGF-1 genes and neuron death, PI3K-Akt, and human papillomavirus infection signalling pathways may possibly explain this association.

### Electronic supplementary material

Below is the link to the electronic supplementary material.

Supplementary Material 1

Supplementary Material 2

Supplementary Material 3

Supplementary Material 4

Supplementary Material 5

Supplementary Material 6

Supplementary Material 7

Supplementary Material 8

## Data Availability

The code utilized in our research can be found in Additional File 8. The availability datasets generated and/or analyzed during the current study are summarized as following: 1. GWAS datasets: Cervical cancer GWAS in discovery cohort are available in GWAS catalog repository, https://www.ebi.ac.uk/gwas/downloads/summary-statistics, Accessed 20 January 2023 [23]. GWAS for six neuropsychiatric disorders, including Parkinson’s disease, Anxiety, Alzheimer’s disease, Mood disorders, Depression, and Alcohol dependence, are available in FinnGen repository, https://r8.finngen.fi/. Accessed 20 January 2023 [25]. GWAS of Bipolar disorder (BD) and Schizophrenia (SCZ), are available in GWAS catalog repository, https://www.ebi.ac.uk/gwas/downloads/summary-statistics, Accessed 20 January 2023 [26]. Autism spectrum disorder (ASD) GWAS are available in GWAS catalog repository, https://www.ebi.ac.uk/gwas/downloads/summary-statistics, Accessed 20 January 2023 [27]. Cervical cancer GWAS in replication cohort are available in GWAS catalog repository, https://www.ebi.ac.uk/gwas/downloads/summary-statistics, Accessed 20 January 2023 [28]. Depression GWAS in replication cohort are available in GWAS catalog repository, https://www.ebi.ac.uk/gwas/downloads/summary-statistics, Accessed 20 January 2023 [29]. 2. Depression related genes and cervical cancer related genes. Diseases specific genes are available in DisGeNET repository, https://www.disgenet.org/; Accessed 25 June 2023 [39], Genecards repository, https://genecards.weizmann.ac.il/v3/; Accessed 25 June 2023 [40], and the uniprot repository, https://www.uniprot.org/; Accessed 25 June 2023 [41].

## Abbreviations

GLOBOCAN: Global Cancer Statistics
HPV: High-risk human papillomavirus
GWAS: Genome-wide association studies
UKB: UK Biobank
BD: Bipolar disorder
SCZ: Schizophrenia
PGC: Psychiatric Genomics Consortium
ASD: Autism spectrum disorder
SNPs: Single Nucleotide Polymorphisms
MHC: Major Histocompatibility Complex
MAF: Minor Allele Frequency
MR: Mendelian randomization
LDSC: Linkage disequilibrium score regression
IVs: Instrument variables
TSMR: Two-Sample Mendelian Randomization
TCGA-CESC: The Cancer Genome Atlas-Cervical squamous cell carcinoma and endocervical adenocarcinoma
GO: Gene ontology
KEGG: Kyoto Encyclopedia of Genes and Genomes
PPI: Protein-protein interaction
MCC: Maximal Clique Centrality
BP: Biological process
CC: Cell component
MF: Molecular function
TNF: Tumor necrosis factor
VEGFA: Vascular endothelial growth factor A
JUN: Transcription factor AP-1
IGF-I: Insulin-Like Growth Factor I
