# Supplementary figures and images for: Relationships between nine neuropsychiatric disorders and cervical cancer: insights from genetics, causality and shared gene expression patterns
Source: BMC Womens Health. 2024 Jul 8;24:394. doi: 10.1186/s12905-024-03234-5 (PMC11229200; doi:10.1186/s12905-024-03234-5)

Supplementary fig.1

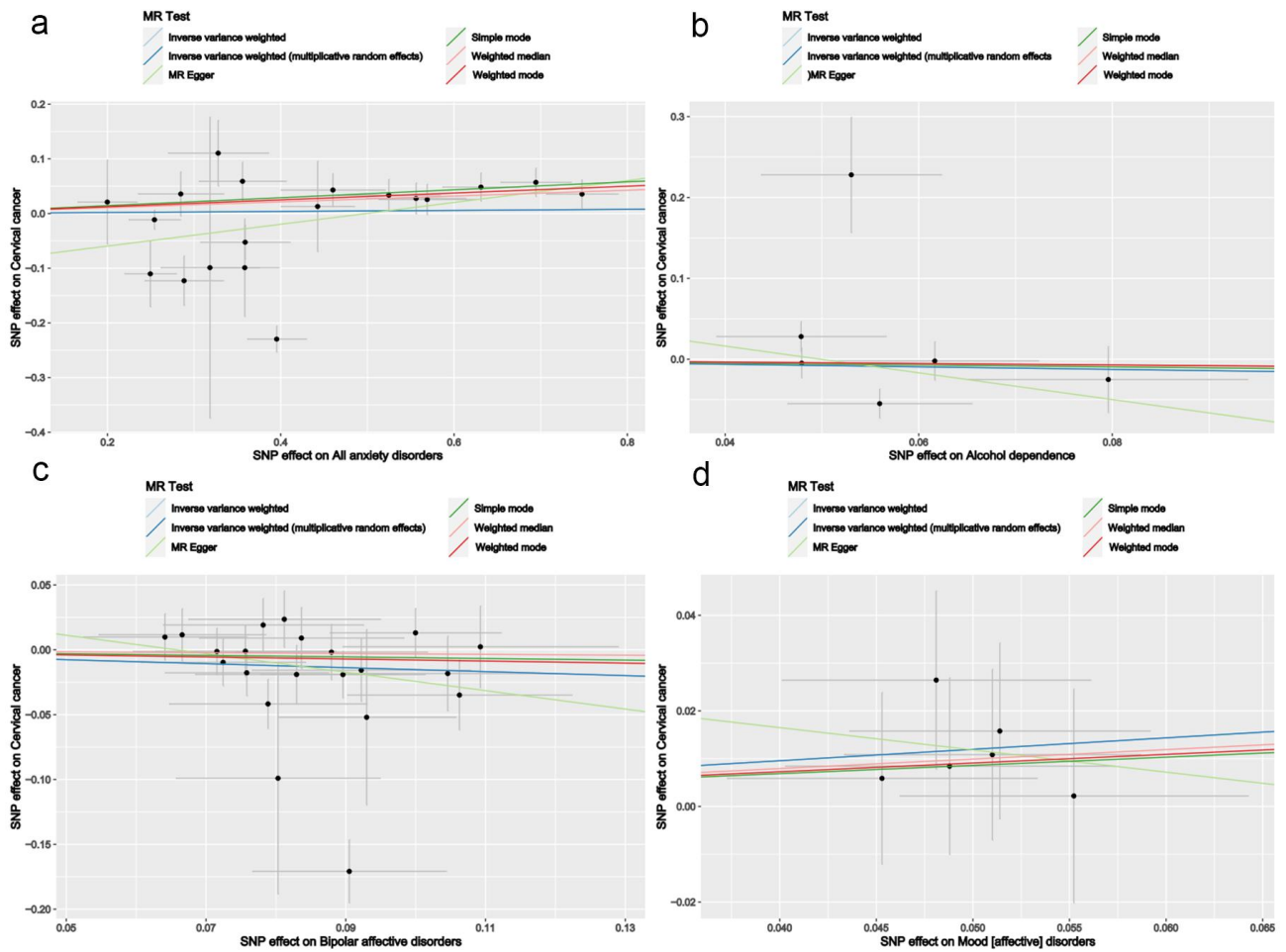

Supplementary fig.2

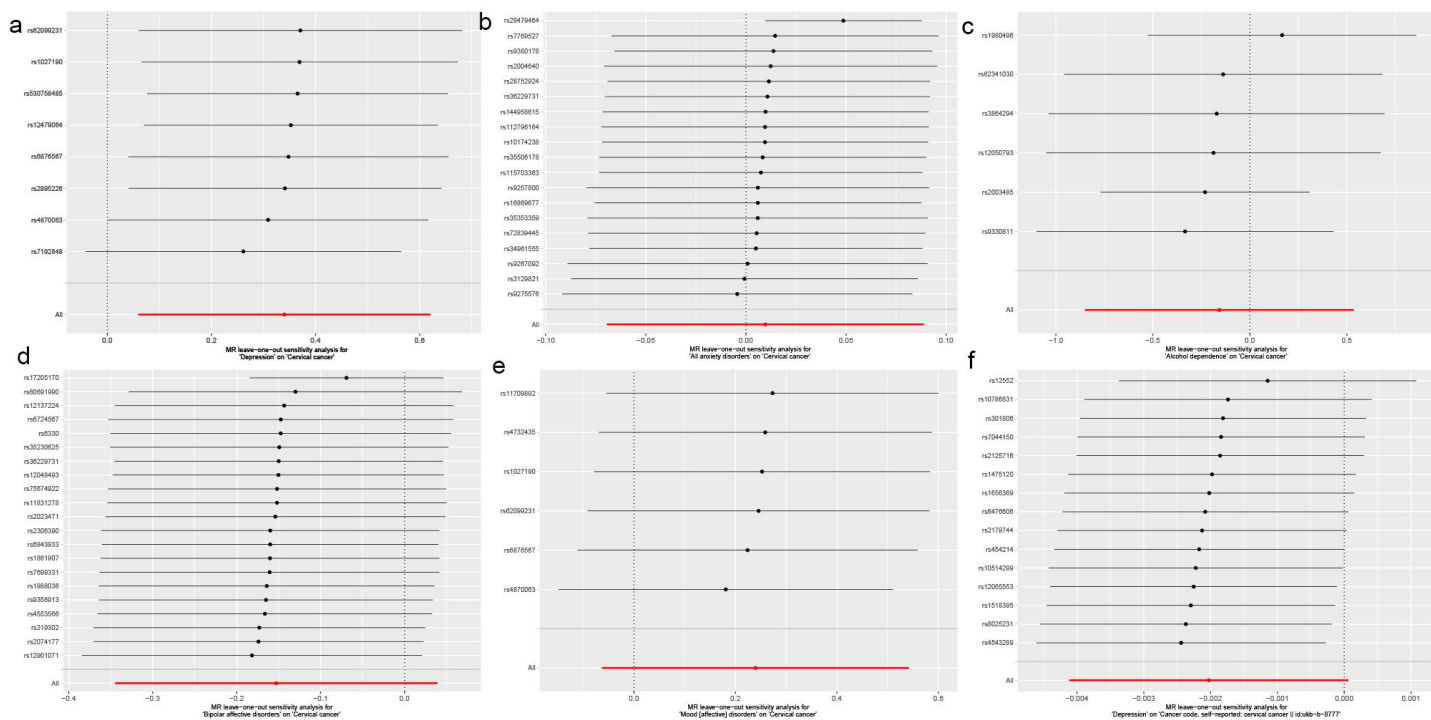

Supplement: Supplementary file 6 — Supplementary Material 6 [file 12905_2024_3234_MOESM6_ESM.pdf]
